# Artificial intelligence in clinical decision-making: a comparison of ChatGPT 5.0 and Gemini 3.0 in otologic cases

**DOI:** 10.1186/s12911-026-03757-x

**Published:** 2026-08-11

**Authors:** Bilge Tuna, Gökhan Tüzemen, Hasan Mutlu

**Affiliations:** Bursa City Training and Research Hospital Otorhinolaryngology Department, Bursa, 16110 Türkiye

**Keywords:** Artificial intelligence, ChatGPT 5.0, Gemini 3.0, AIPI, Otology

## Abstract

**Background:**

Large Lingual Models (LLMs) can suggest treatment options based on a patient’s history and symptoms. However, they may lack the ability to deliver fully patient-specific recommendations, which may lead to misdiagnosis or inappropriate treatment decisions.

**Objective:**

This study compared the diagnostic accuracy, investigation recommendations, and treatment planning consistency of two LLMs, ChatGPT 5.0 and Gemini 3.0, using real-world otologic cases.

**Methods:**

Fifty retrospective cases with diverse clinical characteristics (symptoms, examination findings, audiometric tests, and radiological sections) were selected. Three experienced otorhinolaryngologists established the final diagnoses as a gold standard. The cases were processed by both LLMs, which were instructed to act as ENT specialists. Two blinded reviewers independently evaluated the responses using the Artificial Intelligence Performance Instrument (AIPI) across four domains: summarization, differential diagnosis, additional examination, and therapy options. Inter-rater reliability was assessed via Cohen’s kappa.

**Results:**

Both models demonstrated high performance in managing otologic cases. However, Gemini 3.0 significantly outperformed ChatGPT 5.0 in diagnostic accuracy, investigation/therapy planning, and overall AIPI scores (*p* < 0.05). Subgroup analysis revealed that Gemini 3.0 performed higher AIPI scores in ‘vertigo’ cases, while similar performances were found in ‘otits media’, ‘hearing loss’ and ‘facial paralysis’ groups. Inter-rater agreement for AIPI scores was excellent.

**Conclusion:**

To our knowledge, this is the first study evaluating LLMs using real-world otologic data. While both models show potential as clinical decision-support tools, Gemini 3.0 exhibited superior diagnostic performance in real-world otologic cases. Future research should include larger populations and endoscopic imaging to further validate these findings.

## Introduction

One of the most exciting technological advancements in recent years has been the introduction of artificial intelligence (AI) chatbots based on large language models (LLMs). These AI chatbots are capable of generating human-like text and engaging in conversational interactions. In November 2022, OpenAI publicly released the Chatbot Generative Pre-trained Transformer (ChatGPT), an AI chatbot capable of responding to user queries in a conversational manner and producing outputs that are nearly indistinguishable from those of humans. The release of ChatGPT represents a technological revolution with the potential to transform healthcare as we know it, including hearing health services. ChatGPT is not an isolated development but part of a global race to create increasingly advanced AI chatbots. In addition to OpenAI, Google introduced the Gemini model to the public in 2023, establishing itself as a major competitor in this rapidly evolving field. LLMs, have emerged as powerful tools in medicine, offering significant potential to streamline diagnostic and management workflows and support clinical decision-making processes. Recent studies suggest that advanced LLMs may approach or even surpass human performance in certain medical assessments, including Medical Licensing Examinations and otolaryngology–head and neck surgery board certification exams [1, 2].

The development of artificial intelligence–driven language models such as ChatGPT and Gemini is gaining increasing importance in the field of medicine. Both patients and physicians now have easy and widespread access to chatbots capable of providing medical information. AI chatbots can suggest treatment options based on a patient’s history and symptoms and provide information regarding the benefits and risks associated with each option. However, unlike human healthcare professionals, they may lack the ability to deliver fully patient-specific recommendations, which may lead to misdiagnosis or inappropriate treatment decisions. According to a recent review of applications of multimodal AI in the field of otolaryngology, the most frequently focused subspecialty was cancer (75%), followed by general otolaryngology (11%) [3]. Current otolaryngology literature agrees that AI improves diagnostic accuracy and eases the paperwork/analysis burden on clinicians [4]. However, to our knowledge, the impact of AI on decision-making in otology-related cases has not been previously investigated. The aim of this study is to compare the performance and clinical adequacy of ChatGPT and Gemini in the processes of diagnosis, investigation, and treatment planning in real-world otologic cases.

## Materials and methods

In this study, data from 50 patients with diverse otologic clinical presentations who were treated in our clinic between 2022 and 2025 were retrospectively analyzed. Following a comprehensive review by a council of three otorhinolaryngologists, the definitive diagnoses and treatment processes of all cases were established. The cases were then presented separately in two language models (ChatGPT 5.0 and Gemini 3.0; March 1–3, 2026), including clinical features, audiometric test results, and radiological imaging data. Standardized and comparable prompts were used during all case presentations, which were completed within a three-day period. Both ChatGPT 5.0 and Gemini 3.0 were accessed and evaluated via their official online interfaces. Due to the lack of on-premise AI infrastructure at our institution and in strict compliance with data privacy regulations, a meticulous de-identification protocol was enforced. All clinical narratives, radiological reports, and diagnostic data were completely stripped of personally identifiable information prior to system upload to guarantee absolute patient anonymity. The information included in each prompt was as follows: “age, gender, duration of complaints, comorbidities, family history, physical examination, previous surgical history, prior and current medications, audiometric tests, radiological image sections’’. Each prompt included the following question: “What are the two most likely diagnoses for this case? Think like an experienced ENT specialist and plan the diagnostic examination, treatment, and follow-up processes.” The Table 1 provides an example question illustrating a case of traumatic facial paralysis.

**Table 1:** Example prompt and responses from ChatGPT 5.0 and Gemini 3.0

Case: traumatic facial paralysis	
A 47-year-old female patient presented with complaints of numbness and limited movement on the right side of her face. She had been involved in a traffic accident two months prior, and right peripheral facial paralysis was diagnosed on the fifth day of her stay in the intensive care unit. An electromyography (EMG) performed 45 days after the accident revealed total axonal degeneration of the right facial nerve. The patient has no known underlying medical conditions or familial genetic diseases. On physical examination, she exhibited grade 6 right facial paralysis, while other otorhinolaryngologic findings were normal. Audiometry tests and radiological imaging results are included as supplementary information. What are the two most likely diagnoses for this case? Please outline the investigation, treatment, and follow-up processes as if you were an ENT specialist	
	
**ChatGPT 5.0**	**Gemini 3.0**
1. Most Likely To Diagnose: Traumatic Facial Nerve Injury (Due to Longitudinal Temporal Bone Fracture)	1. Facial Nerve Injury Due to Temporal Bone Fracture (Most Likely Diagnosis)
2. Second possibility: Fallopian canal segmental compression	2. Severe crushing/contusion of the facial nerve (without fracture)
*******************************	******************************
Approach Based on the Most Likely Diagnosis: Traumatic Facial Nerve Injury	Management Recommendations According to the Most Likely First Diagnosis (Facial Nerve Injury Due to Temporal Bone Fracture)
	When total axonal degeneration (Grade VI paralysis and EMG finding) is detected, time is running out for the indication of surgical decompression.
**A) Diagnostic Plan**:	**A. Diagnosis and Diagnostic Development (Already Done and Additions)**
1. High-resolution temporal bone CT (fracture line localization)	1. House-Brackmann Rating: It should continue to be used to regularly monitor the progression and regression of paralysis.
2. Contrast enhanced MRI (nerve segment edema?)	2. Electrophysiologic Tests:
3. Serial EMG (repeat in 3 months)	-Neuroneurography (ENoG): If the nerve response on the paralyzed side is reduced by more than 90% compared to the contralateral side (consistent with the finding of total axonal degeneration), it is an important threshold for the decision to undergo surgical decompression.
**B) Surgical Indication Evaluation**:	-EMG: Nerve repair or grafting may be considered as total axonal degeneration is confirmed.
Surgery is considered in traumatic paralysis:	3. Temporal Bone High-Resolution CT: It is essential to pinpoint the precise location of the fracture line (whether it involves the labyrinth capsule), the presence of bone fragments in the nerve canal, and their potential point of compression on the nerve.
• HB 5–6	**B. Treatment (Surgical Decompression)**
• More than 90% degeneration on EMG	If total axonal degeneration is detected and the paralysis persists for 2 months, a critical time period for the indication of emergency surgery may have been exceeded (usually the first 2-3 weeks). However, if it is radiologically determined that there is an anatomical compression or interruption in the nerve, surgical discovery and decompression are the only options.
• Early life	1. Surgical Decision:
But:	-Indication: Bone compression in the nerve canal or more than 90% degeneration in ENoG is detected on CT (but the value of ENoG is low after 2 months). Total axonal degeneration on EMG indicates that conservative treatment will fail.
This patient is now 2 months after the trauma.	-Procedure: Facial Nerve Decompression (Pressure Removal): Depending on the location of the fracture line, the compression on the nerve is eliminated with a transmastoid, middle fossa, or both combined approach.
Effectiveness of surgical decompression:	-Nerve Repair: If a complete rupture of the nerve (neurothemesis) is detected, nerve grafting (e.g. using the *suralis* nerve) should be performed.
• Higher in the first 2–3 weeks	2. Medical Treatment: Corticosteroids (prednisolone) are often used to reduce edema, but their benefit is limited in a 2-month stroke.
• The benefit decreases after 2 months	**C. Follow-up**
**C) Proposal at the Current Stage**:	1. Physical Therapy: Intensive facial muscle physiotherapy (massage, mimic exercises) should be started immediately to prevent atrophy of the facial muscles and support the healing process.
Eye protection (urgent and priority)	2. Eye Care: Since the paralysis is total, aggressive eye care such as eye closure (tarsorrhaphy), artificial tears and night moisturizing should be applied to prevent corneal drying and damage.
• Artificial tears	3. Long-Term Follow-Up: Recovery after surgery can be slow (may take months). If there is no improvement after 1 year, dynamic reanimation such as nerve transfers (e.g., *hypoglossal* nerve transfer) or static procedures (face lift) may be considered.
• Night closure	
• Temporary tarsorrhaphy if necessary	
Physical therapy / facial exercises	
EMG again at 3 months	
**D) If there is no improvement in 6 months**:	
Reconstruction options:	
• Hypoglossal–facial anastomosis	
• Wired graft	
• Cross-facial nerve graft	
• Dynamic muscle transfer	
Prognosis:	
If there is total axonal degeneration:	
• The probability of spontaneous full recovery is low	
• There may be partial recovery	
• Synkinesia may develop	
Abstract:	
This patient is now out of the acute period.	
Priority:	
1. Eye protection	
2. Serial EMG	
3. Reconstructive planning in 3–6 months	

The responses generated by the language models for each case—including their interpretations, proposed diagnoses, and recommended diagnostic and treatment plans—were evaluated by two blinded otorhinolaryngologists using the Artificial Intelligence Performance Instrument (AIPI) scoring system (Fig. 1). The scores of both language models were subsequently compared.

**Fig. 1:**
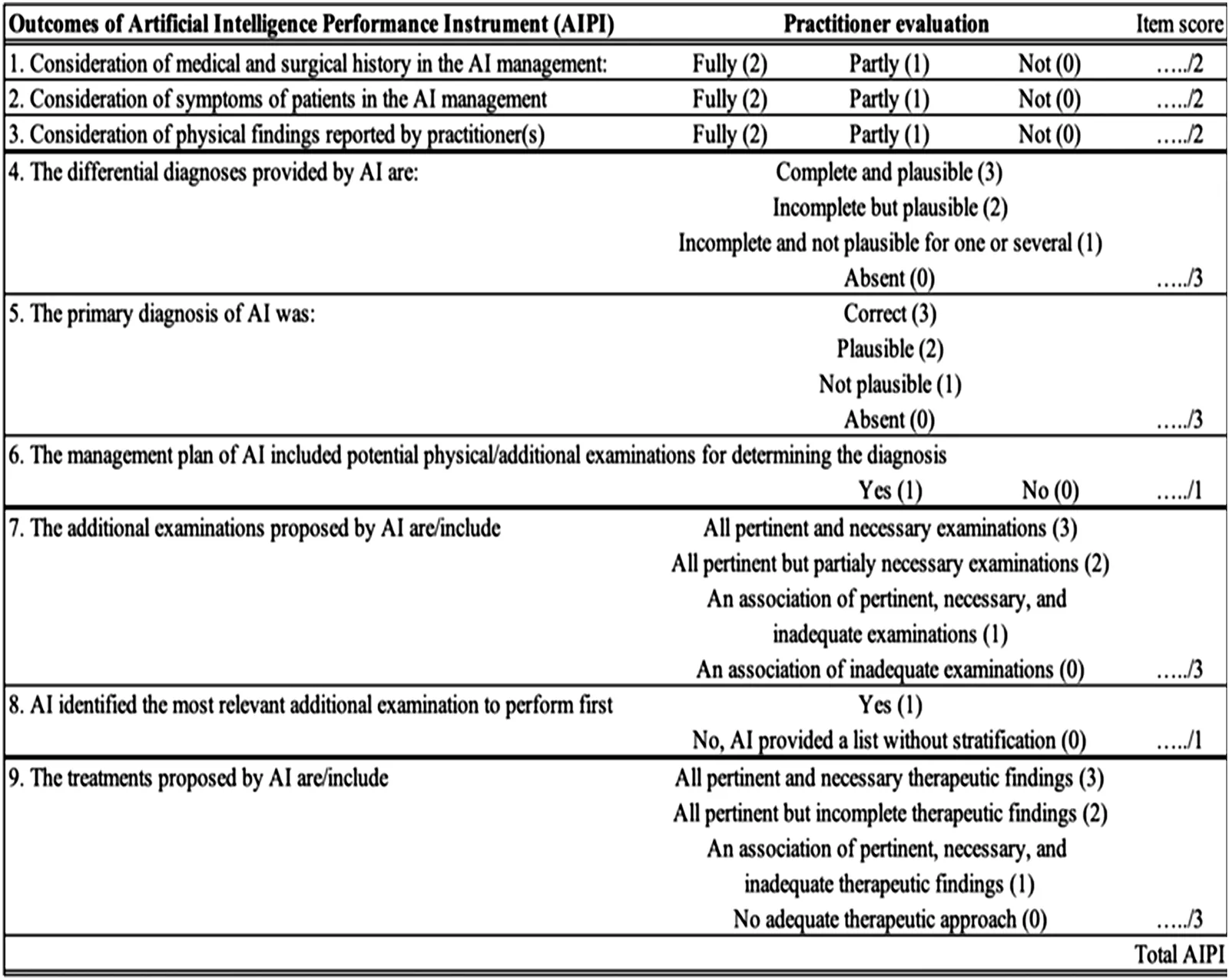
Artificial intelligance performance instrument (AIPI)

The AIPI is defined as a tool for evaluating the performance of artificial intelligence systems [5]. The responses provided by the language models to the prompts were assessed and scored across three main domains: summarization/interpretation of case data, diagnostic suggestions, and recommendations for diagnostic workup and treatment.

AIPI Subdomains:

-Explanation and summarization.

-Diagnostic evaluation (differential diagnosis).

-Diagnostic workup and treatment recommendations.

Scoring Range (Total AIPI Score):

0–5 → Inadequate performance.

6–10 → Low level of appropriateness.

11–15 → Moderate level of appropriateness.

16–20 → High performance.

In our study, cases were additionally categorized into four subgroups based on the predominant presenting symptom, and the performance of the language models was evaluated separately within each group. The grouping was determined according to the patient’s primary presenting complaint. The number of patients in each subgroup was as follows: hearing loss (*n* = 18), otitis (*n* = 17), vertigo (*n* = 10), and peripheral facial paralysis (*n* = 5).

### Distribution of diagnoses within subgroups

#### Hearing loss group (*n* = 18)

Idiopathic tinnitus (*n* = 1), sudden sensorineural hearing loss (*n* = 3), unilateral idiopathic sensorineural hearing loss (*n* = 1), bilateral mild-to-moderate sensorineural hearing loss (*n* = 1), bilateral total sensorineural hearing loss (*n* = 2), conductive hearing loss (*n* = 3), acoustic neuroma (*n* = 5), middle ear neuroendocrine tumor (*n* = 1), ear osteoma (*n* = 1).

#### Vertigo group (*n* = 10)

Meniere’s disease (*n* = 3), vestibular neuritis (*n* = 1), perilymph fistula (*n* = 1), labyrinthitis (*n* = 2), posterior canal benign paroxysmal positional vertigo (BPPV) (*n* = 1), horizontal canal BPPV (*n* = 1), central positional vertigo (*n* = 1).

#### Otitis media group (*n* = 17)

Traumatic tympanic membrane perforation (*n* = 1), middle ear foreign body (*n* = 1), petrous bone granuloma (*n* = 1), non-suppurative chronic otitis media (*n* = 1), chronic serous otitis media (*n* = 5), cholesteatomatous chronic otitis media (*n* = 5), subperiosteal abscess (*n* = 1), chronic otitis media with petrositis (*n* = 1), sinus thrombosis (*n* = 1).

#### Facial paralysis group (*n* = 5)

Temporal bone fracture (*n* = 3), otitis media (*n* = 1), herpes zoster oticus (*n* = 1).

### Statistical analysis

Data were analyzed using SPSS v26.0 and Python 3.10. Normality was assessed via the Shapiro-Wilk test. Since the data were not normally distributed, non-parametric tests were employed. Inter-rater reliability between the two reviewers was calculated using Quadratic Weighted Kappa and interpreted according to Landis and Koch’s criteria. Comparisons between the LLMs for all clinical parameters (summarization, diagnosis, additional examinations-therapy options, and total AIPI scores) were performed using the Wilcoxon Signed-Rank Test. Differences across clinical subgroups (Otitis, Vertigo, Hearing Loss, and Facial Paralysis) were evaluated with the Kruskal-Wallis H test. To control the family-wise error rate due to multiple comparisons across clinical subgroups and parameters, p-values were adjusted using the Holm-Bonferroni method. Results with an adjusted p-value of < 0.05 were considered statistically significant.

## Results

Both chatbots demonstrated high performance in total AIPI scoring (maximum score: 20); however, Gemini showed significantly better performance than ChatGPT (ChatGPT 5.0: mean 18.22 vs. Gemini 3.0: mean 19.07; *p* = 0.002).

In the diagnostic domain (maximum score: 7), Gemini also outperformed ChatGPT (6.68 vs. 6.28, respectively; *p* < 0.001). Both chatbots demonstrated excellent ability to analyze clinical features and provided case information (maximum score: 6), with no statistically significant difference between the models (ChatGPT 5.0: mean 5.87; Gemini 3.0: mean 5.84; *p* = 0.317) (Table 2; Fig. 2).

**Table 2:** Cohen’s kappa coefficient was used to measure inter-rater reliability

Sub-groups	scales	ChatGPT 5.0 (med ± SD)	Gemini 3.0 (med ± SD)	*p* value
**All cases** (*n* = 50)	summarization	5.87 ± 0.49	5.84 ± 0.47	0.317
	diagnosis	6.28 ± 1.50	6.68 ± 1.01	**< 0.001**
	additional examinations therapy options	6.06 ± 1.92	6.55 ± 1.24	**0.006**
	Total AIPI Skoru	18.22 ± 3.61	19.07 ± 2.49	**0.002**
**Vertigo**	summarization	6.00 ± 0.00	6.00 ± 0.00	1.000
(*n* = 10)	diagnosis	5.90 ± 1.45	6.80 ± 0.52	0.021*
	additional examination and therapy options	5.60 ± 2.44	6.85 ± 0.49	0.048*
	Total AIPI	17.55 ± 3.78	19.65 ± 0.75	0.036*
**Otitis media**	summarization	5.85 ± 0.36	5.82 ± 0.39	0.564
(*n* = 17)	diagnosis	5.97 ± 1.95	6.47 ± 1.48	0.014*
	additional examination and therapy options	5.71 ± 2.33	6.21 ± 1.72	0.093
	Total AIPI	17.53 ± 4.51	18.50 ± 3.40	0.059
**Hearing loss**	summarization	5.78 ± 0.72	5.78 ± 0.64	1.000
(*n* = 18)	diagnosis	6.64 ± 1.07	6.72 ± 0.78	0.257
	additional examination and therapy options	6.50 ± 1.21	6.58 ± 1.11	0.671
	Total AIPI	18.92 ± 2.86	19.08 ± 2.38	0.436
**Facial paralysis**	summarization	6.00 ± 0.00	5.80 ± 0.42	0.157
(*n* = 5)	diagnosis	6.80 ± 0.63	7.00 ± 0.00	0.317
	additional examination and therapy options	6.60 ± 0.70	7.00 ± 0.00	0.103
	Total AIPI	19.40 ± 0.84	19.80 ± 0.42	0.103

**Fig. 2:**
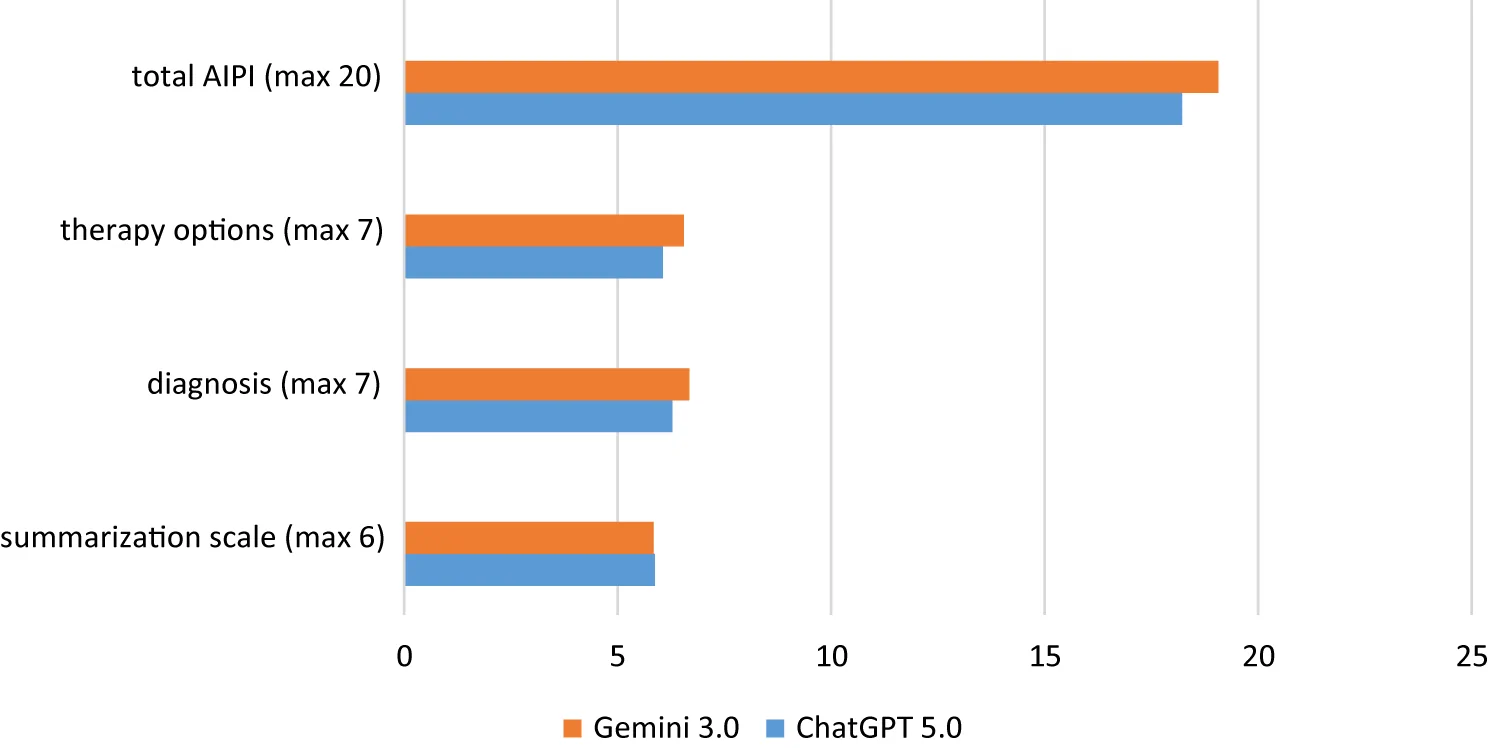
Artificial intelligance performance instrument (AIPI) scores for Gemini 3.0 and ChatGPT 5.0 (Each bar is the average of the two independent reviewers grading)

The mean scores of all 50 cases, as evaluated by the two reviewers, are presented in the figure (Fig. 3).

**Fig. 3:**
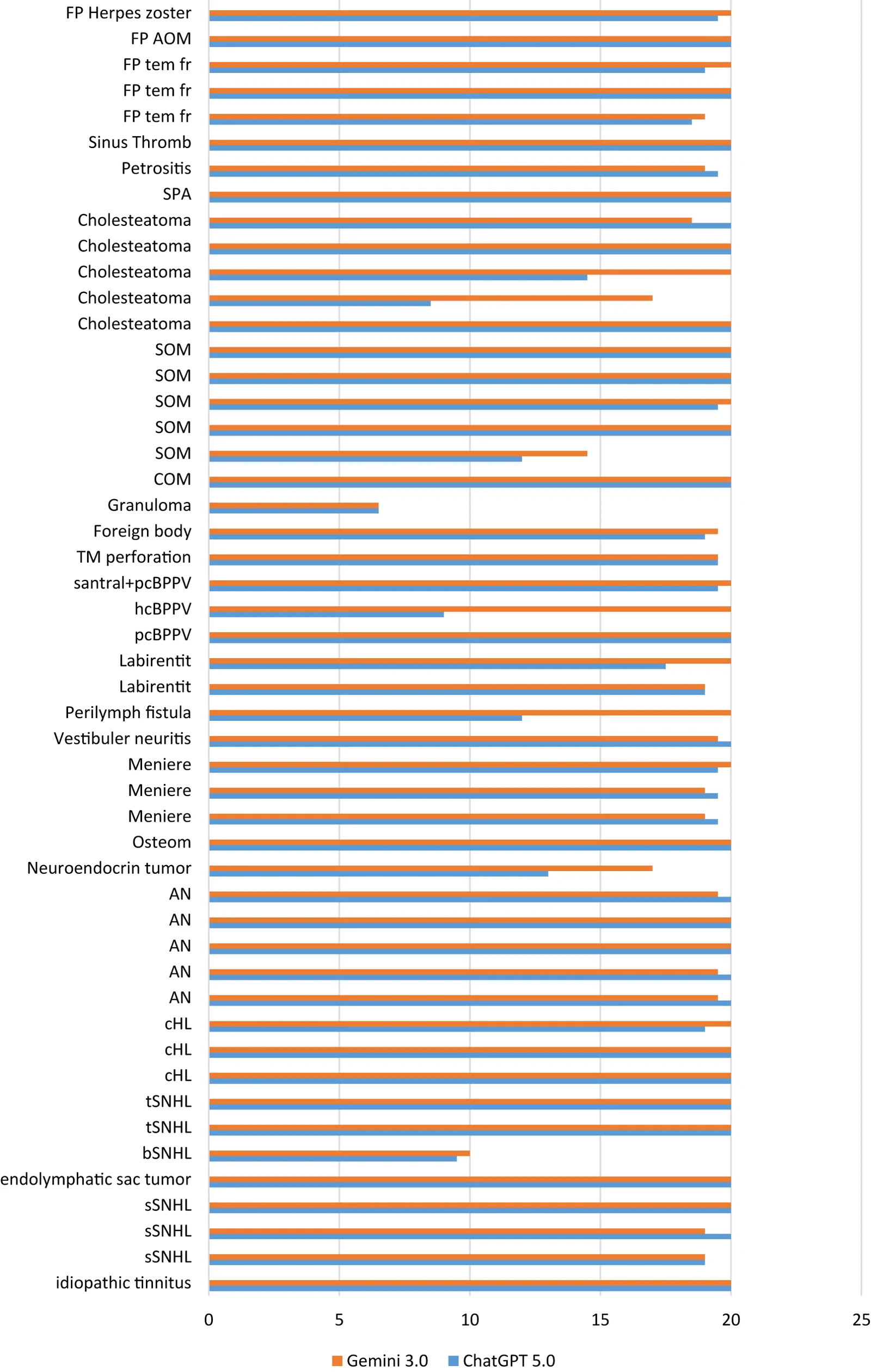
Performance of Gemini 3.0 and ChatGPT 5.0 for each case (Each bar is the average of the two independent reviewers grading)

In subgroup analysis, Gemini was found to have higher AIPI scores in cases of vertigo, whereas similar performances were observed between the two language models in ottis media, hearing loss and facial paralysis groups (*p* = 0.036, 0.059, 0.436, and 0.103, respectively) (Table 2).

Inter-rater reliability was assessed using Cohen’s kappa statistics and the analysis demonstrated almost perfect agreement for total AIPI scores. Inter-rater reliability between the two independent reviewers was assessed using Quadratic Weighted Cohen’s Kappa to account for the ordinal nature of the scoring systems. The results demonstrated substantial to almost perfect agreement across all evaluated parameters (Table 3).

**Table 3:** Cohen’s kappa coefficient was used to measure inter-rater reliability

	Quadratic weighted Cohen’s Kappa	95% confidence interval	compatibility (Interpretation)
Summarization			
ChatGPT 5.0	0.784	0.71–0.85	(Substantial)
Gemini 3.0	0.722	0.65–0.79	(Substantial)
Diagnosis			
ChatGPT 5.0	0.928	0.88–0.97	(Almost Perfect)
Gemini 3.0	0.876	0.81–0.94	(Almost Perfect)
Additional examination and therapy options			
ChatGPT 5.0	0.940	0.90–0.98	(Almost Perfect)
Gemini 3.0	0.889	0.82–0.95	(Almost Perfect)
Total AIPI			
ChatGPT 5.0	0.953	0.92–0.98	(Almost Perfect)
Gemini 3.0	0.871	0.80–0.94	(Almost Perfect)

## Discussion

To the best of our knowledge, this is the first study in the field of otology to compare the performance of two prominent LLMs in clinical decision-making using real-world patient data. Both models demonstrated high performance, supporting their potential role as clinical decision-support tools in otology. In terms of interpreting patient history and clinical information, ChatGPT 5.0 achieved full scores in 88% of cases, while Gemini 3.0 achieved full scores in 84%. Both models showed a strong ability to analyze clinical features and provided information accurately in the majority of cases. Their responses were consistent in the interpretation of audiometric test results, and both models demonstrated comparable performance in the evaluation of radiological imaging.

Regarding diagnostic performance, ChatGPT 5.0 provided completely accurate diagnostic suggestions in 72% of cases, whereas Gemini 3.0 achieved this in 82% of cases. In terms of diagnostic workup and treatment planning, the proportion of cases receiving full scores was 64% for ChatGPT 5.0 and 78% for Gemini 3.0.

Subgroup analyses revealed that Gemini 3.0 demonstrated higher AIPI scores in vertigo cases and, both models performed similarly and achieved similar AIPI scores in otitis media, hearing loss and facial paralysis groups. However we should point the fact that the cases are less sufficient in each subgroups which weekened the confidence. Conducting multicenter prospective clinical trials and increasing the number of cases in heterogeneous groups with more diverse case types would yield more reliable results in this area.

Across all groups, the lower standard deviations observed in the Gemini 3.0 model compared to ChatGPT 5.0 suggest that Gemini 3.0 produces more consistent outputs in clinical decision-support processes. This finding indicates a higher level of reliability and stability in its responses.

It is important to note that the responses of language models may vary over time. In a study designed to evaluate the accuracy and reproducibility of ChatGPT 4.0, responses to 30 anonymized hearing-related questions demonstrated an agreement rate of 85–88% across different days [6]. To minimize potential variability in responses across time, all case presentations to the language models in our study were completed within a three-day period.

In another study, the ChatGPT 4.0 model was tested on a dataset of 1,000 simulated cases that included monthly hearing threshold measurements over a 12-month period. The findings demonstrated that the model has significant potential as a clinical decision-support tool for interpreting hearing tests and tracking longitudinal changes over time [7]. More recently, a study comparing ChatGPT and Gemini performance on 80 simulated audiograms reported that the ChatGPT-5.0 Plus model demonstrated superior accuracy and consistency in interpreting pure-tone audiograms compared to Gemini 2.5 [8]. In our study, both language models demonstrated high performance and consistent interpretations in the analysis of audiometric test results, supporting their potential utility in audiological assessment.

Previous studies have reported that the ChatGPT model demonstrates high performance in clinical summarization and interpretation tasks [9, 10]. In our study, ChatGPT 5.0 achieved slightly higher scores in explanation, recommendation, and summarization domains. This finding suggests that ChatGPT 5.0 may serve as an effective tool for patient education and for facilitating shared decision-making processes in clinical practice.

Diagnostic accuracy represents one of the key strengths of LLMs. Prior studies have indicated that both ChatGPT and Gemini are capable of providing accurate primary diagnoses even in complex medical cases [10, 11]. Consistent with the literature, both models demonstrated high diagnostic performance in our study.

In another study comparing treatment recommendations for primary head and neck cancers, the ChatGPT model and the Gemini Advanced model were evaluated, with ChatGPT reported to have a slight advantage in this domain [12]. As otologic malignancies were not included in our study, further research focusing on such cases would be valuable to enable more comprehensive clinical comparisons in this area.

Many aspects of otolaryngology practice have the potential to be revolutionized by generative AI, including the preparation of the consultation, the identification of potential emergency, the analysis of mucosa lesions, and the improvement of research ideas and protocols [13]. Our study indicates that, in the field of otology as well, AI will play a significant role in the clinical decision-making process for clinicians. Our study specifically focuses on the diagnostic and therapeutic journey of patients presenting with complex otological conditions. The methodology of our research is unique: we provided the LLMs with detailed medical histories and physical examination findings, and subsequently challenged the models to interpret audiometric test results and radiological imaging findings. By simulating a real-world clinical workflow, we evaluated the models’ ability to synthesize multimodal clinical data—a critical step toward integrating AI into specialized medical practice. This study serves as a benchmark for the current capabilities of AI in our field and highlights the essential areas for further development to ensure LLMs can function as reliable clinical decision-support tools. We believe our work provides a foundation for the safe and effective integration of AI in otology.

As a tertiary care hospital in Turkey, we predominantly implement established Western guidelines alongside localized treatment protocols in our clinical practice. However, AI-based decision support systems seems to heavily reflect high-resource Western standards, posing adaptation challenges for institutions balancing local socioeconomic and epidemiological realities. To bridge this gap, the World Health Organization (WHO) and recent literature emphasize three core strategies: (a) local adaptation of global models using regional datasets, (b) collaborative training to build balanced AI systems across multiple centers without sharing raw patient data, and (c) clinical oversight to treat AI outputs as supportive second opinions rather than absolute truths [14, 15].

The rapid evolution of LLMs versions and the related changes in the landscape underscore the need for ongoing evaluation [13]. We would like to illustrate some areas where ChatGPT 5.0 model lost score points in our study. For instance, in a patient diagnosed with horizontal canal BPPV, the ChatGPT 5.0 failed to recommend evaluation of positional nystagmus and appropriate repositioning maneuvers. In a case of Meniere’s disease, it did not suggest relevant objective diagnostic tests. In a patient with facial paralysis resulting from a temporal bone fracture, decompression was not recommended as a treatment option despite total axonal degeneration detected in the electromyography performed at two months (it was not considered effective after the second or third week). However, following an evaluation at our clinic, a surgical decision was made for the same patient, and facial decompression surgery was performed. In the postoperative period, the patient’s hearing was preserved, and facial functions improved (*see* Table 1). Similarly, the model did not recommend surgical exploration for a case of perilymph fistula and also failed to mention the need for a biopsy in a patient with a suspected middle ear neuroendocrin tumor.

We would also like to give examples of some sections where both language models lost score points. Examples of deficiencies observed in both language models include the omission of hyperbaric oxygen therapy as a treatment option in patients with sudden sensorineural hearing loss, and failure to recommend surgical exploration in cases with a high suspicion of cholesteatoma based on computed tomography or magnetic resonance imaging findings. Additionally, in a patient suspected of having a petrous bone cholesterol granuloma based on imaging and clinical evaluation, as well as in another patient with bilateral idiopathic sensorineural hearing loss, both models proposed unlikely diagnoses.

Despite these shortcomings, both language models demonstrated high performance in interpreting radiological imaging, particularly in otologic tumor cases. Furthermore, both models achieved full scores in treatment planning in the majority of cases involving chronic otitis complications, otosclerosis, Ménière’s disease, vestibular neuritis, posterior canal BPPV, and acoustic neuroma.

In the future, AI may play an increasingly important role in supporting clinical decision-making in otologic cases. One of its greatest advantages lies in its ability to integrate and synthesize the entirety of available knowledge, including the most recent scientific evidence. As language models continue to evolve, it is conceivable that more advanced systems will emerge, potentially transitioning from supportive tools to foundational guides in clinical decision-making. Integrating AI into clinical workflows faces major hurdles regarding data protection and seamless electronic health record integration. To secure multimodal diagnostic data safely, establishing local, high-security, on-premise AI platforms is essential. In our study, while an online AI model was utilized, strict privacy protocols were maintained by thoroughly de-identifying all patient records and clinical data prior to upload to ensure absolute confidentiality.

Nevertheless, the responsibility of critically evaluating AI-generated recommendations in the context of individual clinical findings will remain with clinicians. Despite continuous improvements in the performance of LLMs, the phrase “There is no disease, there is a patient”, highlighting the importance of individualized patient care, a principle rooted in the Hippocratic tradition and later emphasized by William Osler, who stated that ‘the great physician treats the patient who has the disease.’ [16]. Furthermore, the inherently variable course of diseases across individuals limits the extent and timing to which language models can fully replace clinical judgment. In this context, further research is essential to better understand both the potential risks and benefits of AI chatbots and to ensure their ethical, effective, and safe integration into hearing healthcare services [1].

The tendency of language models to rely solely on the information provided, without actively requesting additional clinical details, underscores the importance of supplying comprehensive and clinically complete data during case presentation to enhance diagnostic accuracy. When sufficient clinical information is available, language models can support clinicians by synthesizing complex clinical scenarios and integrating insights from a vast body of medical literature. Nevertheless, further refinement and human oversight remain essential to ensure safe and reliable use. A critical review in otolaryngology reveals that while 55% of AI studies focus on diagnosis, only 5% address prognosis. Consequently, literature emphasizes a clinician-centered framework where AI outputs are treated not as absolute truths, but as provisional, testable hypotheses to safeguard clinical reasoning [17]. Although LLMs can assist in diagnosing rare diseases, clinicians must be aware of their key limitations, particularly model overfitting and AI hallucinations. Therefore, AI outputs must be treated as preliminary second opinions that require strict clinical validation to ensure patient safety.

The limitations of our study include the absence of endoscopic imaging in case presentations, reliance on clinician-reported examination findings, and the exclusion of otologic malignancies. Future studies should incorporate endoscopic imaging and investigate larger and more heterogeneous patient populations to further validate and expand upon these findings. Additionally, while our total sample size provides a robust benchmark, the patient counts within certain clinical subgroups are relatively small, which limits the statistical power of the subgroup comparisons. These specific results should be considered as exploratory trends awaiting validation in larger multi-centric cohorts.

## Conclusion

This study represents, to our knowledge, the first analysis of the decision making capacity of LLMs in otologic cases. Both models demonstrated high performance and yielded comparable treatment recommendations, supporting their potential role as clinical decision-support tools in otology. Notably, Gemini 3.0 exhibited superior performance compared to ChatGPT 5.0. It is evident that AI models are in a state of continuous development and evolution, with performance improvements observed following successive updates and model advancements. To better guide and contribute to this progress, future studies should incorporate endoscopic imaging and evaluate larger and more heterogeneous patient populations with multicenter integration.

## Data Availability

Availability of data and materials Not Applicable (All clinical cases used in this study were patients who underwent examination and treatment planning at our hospital).

## Abbreviations

LLMs: Large lingual models
AIPI: Artificial ıntelligance performance ınstrument
ChatGPT: Chatbot generative pre-trained transformer
sSNHL: sudden sensorineural hearing loss
bSNHL: bilateral SNHL
tSNHL: total SNHL
cHL: conductive hearing loss
AN: Acoustic neuroma
pcBPPV: posterior canal benign paroxysmal peripheric vertigo
hcBPPV: horizontal canal BPPV
COM: Chronic otits media
SOM: Serous OM
AOM: Acute OM
SPA: Subperiosteal abscess
FP: Facial paralysis
Temp fr: Temporal fracture
